# Evaluating optimal patient-turning procedures for reducing hospital-acquired pressure ulcers (LS-HAPU): study protocol for a randomized controlled trial

**DOI:** 10.1186/s13063-016-1313-5

**Published:** 2016-04-06

**Authors:** David Pickham, Betsy Ballew, Kristi Ebong, Julie Shinn, Mary E. Lough, Barbara Mayer

**Affiliations:** General Medical Disciplines, Stanford Medicine, Menlo Park, CA USA; Office of Research, Patient Care Services, Stanford Health Care, Stanford, CA USA; Digital Health, Stanford Health Care, Stanford, CA USA; Patient Care Services, Stanford Health Care, Stanford, CA USA

## Abstract

**Background:**

Pressure ulcers are insidious complications that affect approximately 2.5 million patients and account for approximately US$11 billion in annual health care spending each year. To date we are unaware of any study that has used a wearable patient sensor to quantify patient movement and positioning in an effort to assess whether adherence to optimal patient turning results in a reduction in pressure ulcer occurrence.

**Methods/design:**

This study is a single-site, open-label, two-arm, randomized controlled trial that will enroll 1812 patients from two intensive care units. All subjects will be randomly assigned, with the aid of a computer-generated schedule, to either a standard care group (control) or an optimal pressure ulcer-preventative care group (treatment). Optimal pressure ulcer prevention is defined as regular turning every 2 h with at least 15 min of tissue decompression. All subjects will receive a wearable patient sensor (Leaf Healthcare, Inc., Pleasanton, CA, USA) that will detect patient movement and positioning. This information is relayed through a proprietary mesh network to a central server for display on a user-interface to assist with nursing care. This information is used to guide preventative care practices for those within the treatment group. Patients will be monitored throughout their admission in the intensive care unit.

**Discussion:**

We plan to conduct a randomized control trial, which to our knowledge is the first of its kind to use a wearable patient sensor to quantify and establish optimal preventative care practices, in an attempt to determine whether this is effective in reducing hospital-acquired pressure ulcers.

**Trial registration:**

ClinicalTrials.gov, NCT02533726.

## Background

Pressure ulcers are insidious complications that affect approximately 2.5 million patients and account for approximately US$11 billion in annual health care spending each year [1]. Acutely ill patients are at risk for the development of pressure ulcers due to immobility, reduced perfusion, and prolonged duration of mechanical ventilation [2]. In 2008 the Centers for Medicare and Medicaid Services discontinued reimbursement for facility-acquired pressure ulcers (also known as hospital-acquired pressure ulcers, HAPUs), as these are considered an avoidable complication often described as a “never event.” Given the tremendous burden that pressure ulcers place on the individual patient and the health care system, there is a substantial need for improved prevention methods [3, 4].

Pressure ulcers form when there is sustained pressure, predominately over bony prominences such as the sacrum, heels, occiput, and shoulders. Unrelieved pressure causes compression of cellular tissue, impaired blood flow, and can lead to localized tissue damage and cellular death. Pressure ulcers initially appear as areas of reddened skin but can quickly develop into large open wounds if the pressure is not relieved.

To prevent pressure ulcers, the currently accepted standard of care is to turn patients at least every 2 h, day and night. However, there are no published research studies that support the every 2-h turning schedule in critically ill patients. Notwithstanding, studies have estimated that compliance with patient-turning protocols are around 60 % and that a significant number of patients are not being turned as frequently as recommended [5, 6]. In intensive care units (ICUs), compliance to turning protocols is even lower, ranging from 38 to 51 % [7, 8]. Potential explanations for this low compliance include low prioritization of turning, difficulty in monitoring a patient’s position, ineffective turn reminders/alerts, and sub-optimal caregiver staffing ratios – all of which hinder efforts to prevent pressure ulcers.

To improve adherence to turning as a preventative practice, Leaf Healthcare Inc. (Pleasanton, CA, USA) has developed a patient-monitoring system designed to optimize patient-turning practices. This system is composed of a small, single, wearable patient sensor that adheres to a patient’s chest, similar to a standard telemetry electrode. The sensor communicates wirelessly to a central monitoring station about the patient’s current position (upright, supine/back, right side, left side) and time-to-next-turn. Data for all patients are displayed on a User-Dashboard, allowing staff to easily identify patients who are in need of turning. The system also allows caregivers to identify restricted positions due to existing pressure ulcers or surgical wounds, as well as to create personalized turning protocols, by varying the degree of turn angle and/or tissue decompression thresholds.

Two single-center clinical studies have been completed using the monitoring equipment. A small first-in-human Institutional Review Board (IRB)-approved study was performed to test the feasibility of the patient sensor, mesh network, and monitoring system, and to collect baseline data regarding turning protocol compliance. After this, a second single-center IRB-approved clinical study tested the efficacy, usability, and safety of the monitoring system (ClinicalTrials.gov #NCT02005692). After implementation of the system, compliance to patient turning was reported to have increased significantly from 64 to 98 % [9].

To date, the study team are unaware of any study that has used a wearable patient sensor to quantify patient movement and positioning, in an effort to assess whether adherence to optimal patient turning results in a reduction in pressure ulcer occurrence. As such, the following protocol describes the study to be conducted to evaluate whether optimal patient turning, defined as regular turning every 2 h with at least 15 min of tissue decompression, reduces HAPUs in acutely ill patients.

## Methods/design

### Design

This study is a single-site, open-label, two-arm, randomized controlled trial. As this is an open-label trial, a short observation pilot study will be undertaken during installation and testing of the patient-monitoring system to account for potential observer bias (the Hawthorne effect). The true intent of the technology will be obfuscated to patients and clinical staff in an effort to record baseline data that more accurately represent current turning practices within these units. These pilot data will also be used to determine minimum turning thresholds for the main study and to assess the representativeness of the main study’s control group. A convenient sample of 25 subjects will be enrolled from each participating unit.

### Randomization

**Fig. 1:**
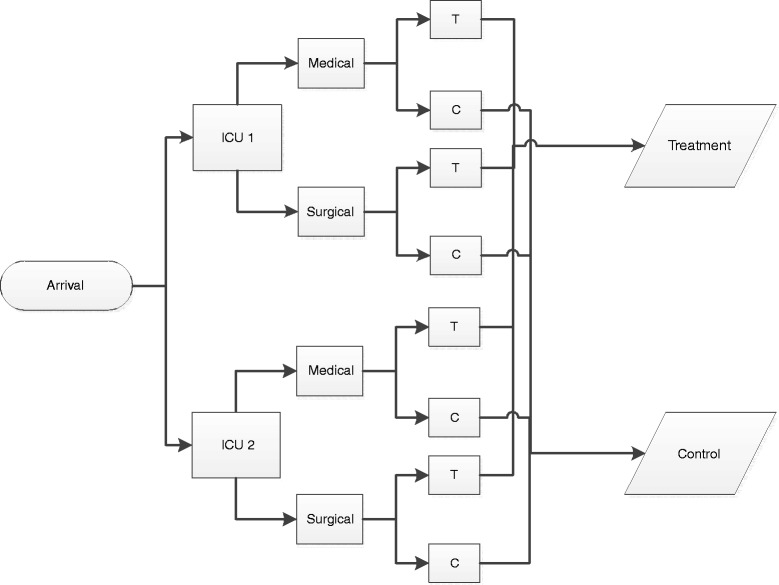
Randomization schema. Stratification by unit and admitting service team

All subjects will be randomly assigned, with the aid of a computer-generated schedule, to receive either standard care (control group) or optimal pressure ulcer preventative care (treatment group). To minimize the risk of predicting the treatment assignment, randomization is performed in permuted blocks of two, four, and six, with random variation of block sizes. To minimize bias at the patient level, randomization is further stratified by unit of admission and admitting service, either medicine or surgery (Fig. 1). Once randomized, each subject will receive a nominal study identification number based on the unit of admission and admitting service. The lowest available number will be provided to each subject in sequential order.

### Study population

This study will enroll all patients admitted to two ICUs. These patients are critically ill with exacerbations of acute and chronic medical conditions, or are receiving aggressive post-surgical care for neurological, cardiac, or trauma-related conditions. They typically have altered levels of consciousness and are dependent on clinical staff for activities of daily living. Due to immobility and other clinical factors these patients are at high risk for developing pressure ulcers. As it is infeasible to gain written consent from this patient population and, due to the minimal risk of the study procedures, a waiver of individual authorization has been granted by Stanford University’s IRB (see Ethics approval section). Therefore, all patients over the age of 18 years admitted to one of the two ICUs will be enrolled in the study. Patients under 18 years of age, those with a known allergy to skin adhesive, or who possess a physical barrier preventing the application of the monitoring sensor, are not eligible for inclusion.

### Recruitment/Intervention

**Fig. 2:**
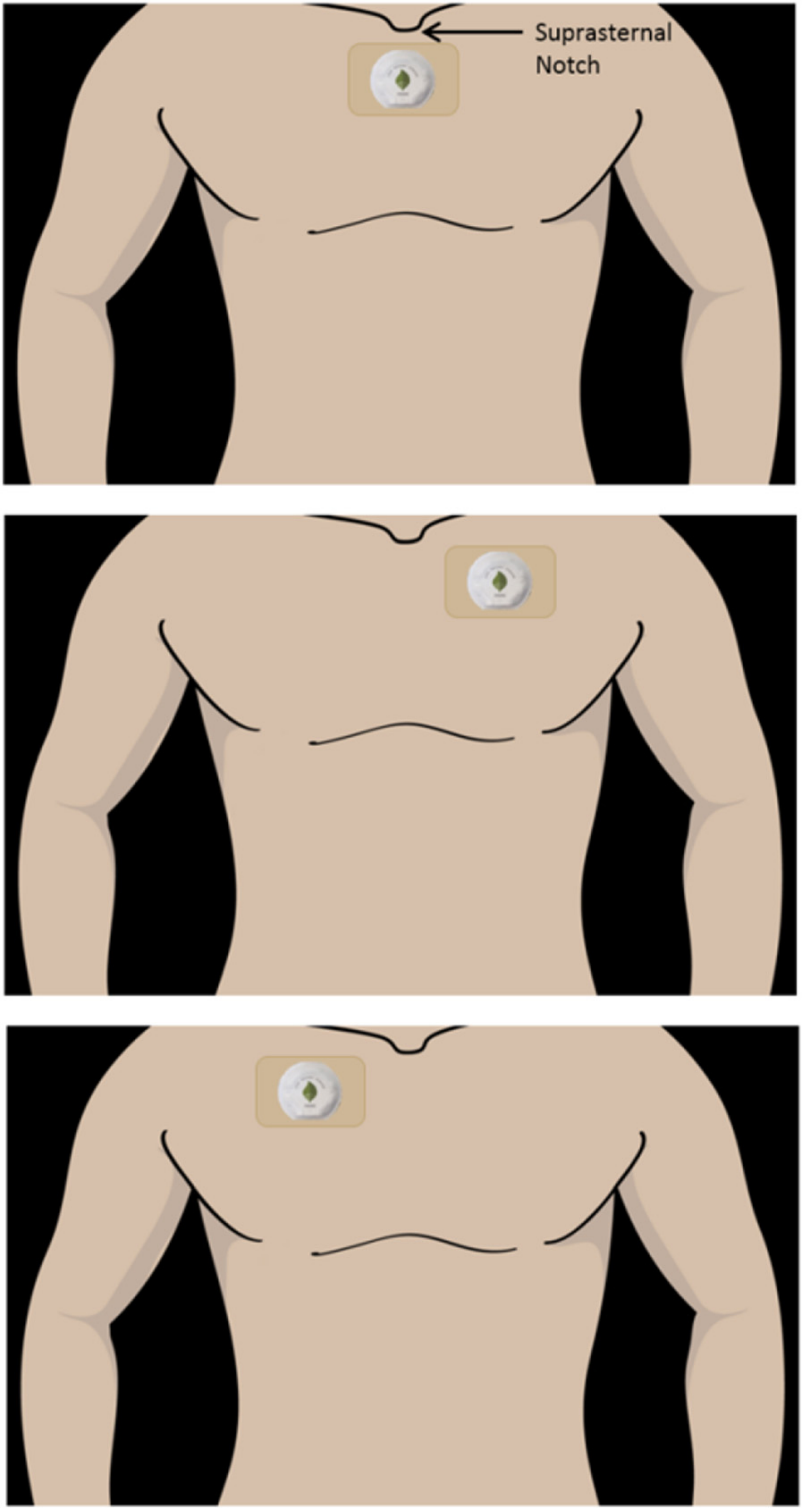
Location of patient-monitoring sensor. *Used with permission from Leaf Healthcare, Inc.

Upon admission to a study unit and as part of standard care, the patient receives a complete “head-to-toe” assessment of their skin. This is performed by two registered nurses (RNs). Any pre-existing pressure ulcer that is detected during this assessment is documented in the electronic medical record (EMR). After initial standard admission procedures are performed, and within the first hour of arrival, nursing staff will place a patient sensor (Leaf Healthcare, Pleasanton, CA, USA) in a predefined location on the patient’s chest (Fig. 2). If an exclusion condition is present, this will be documented and the patient will not be enrolled in the study and will not receive a patient sensor.

For patients included in the study, the unit secretary will provide nursing staff with a patient sensor after associating the sensor’s serial number with the patient’s specific information. This is automatically derived from an Admission-Discharge-Transfer (ADT) data stream communicating directly with the Leaf Patient Monitoring System. At this time the unit secretary will open an envelope, pre-filled with the computer-generated randomized group allocation. A patient sticker will be affixed to the back of the randomization card and stored in a secure location for later retrieval and verification of correct randomization and enrollment. The patient will then be enrolled in either a treatment or control group.

**Fig. 3:**
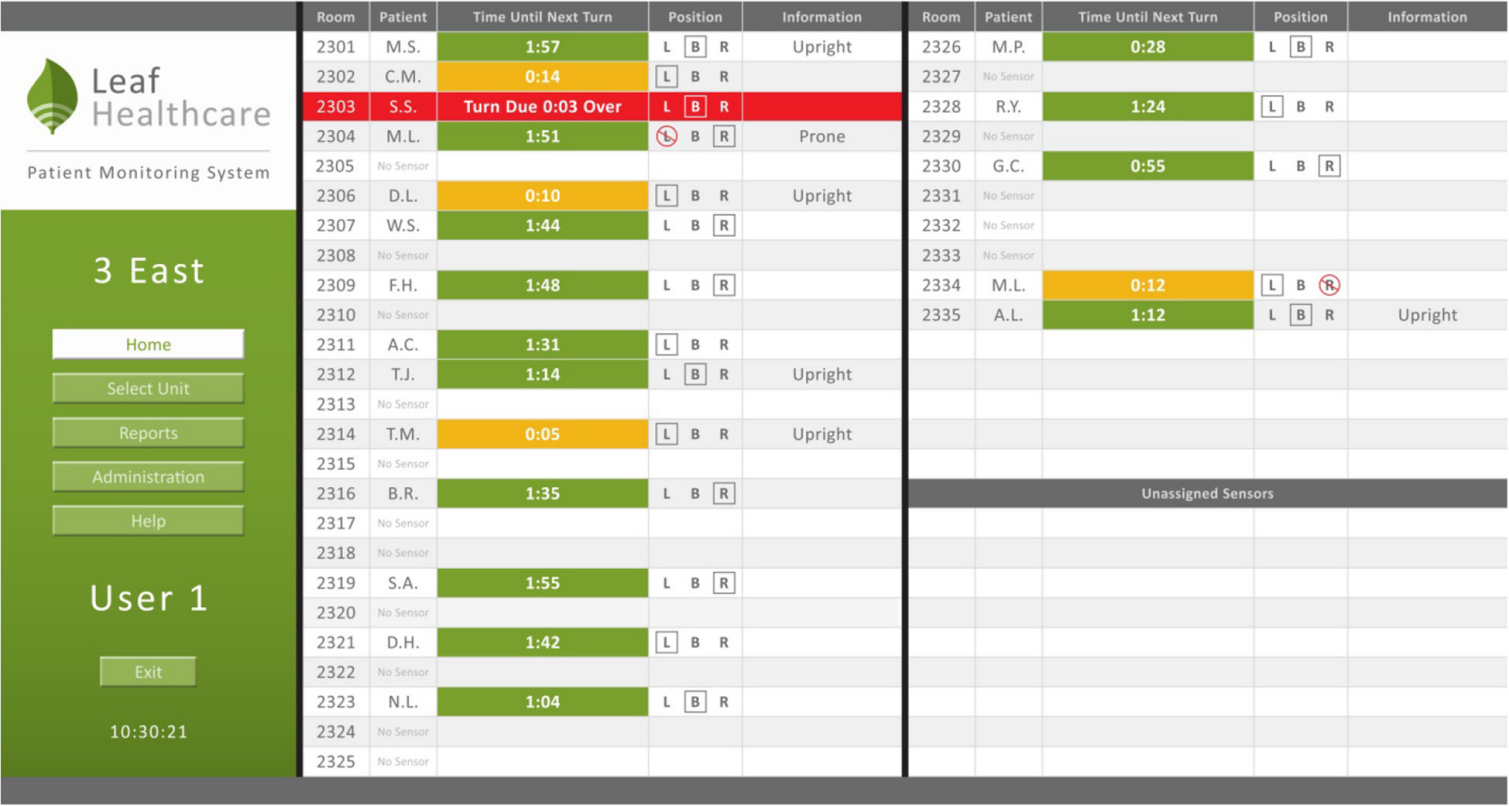
User-Dashboard – Leaf Patient Monitoring Interface. *Used with permission from Leaf Healthcare, Inc.

When a subject is enrolled in the treatment group, the User-Dashboard will be turned “on,” allowing the sensor to communicate the patient’s position and movements (Fig. 3). The clinical team will review this information and be guided by visual displays to provide pressure ulcer-prevention turning practices. With the use of the User-Dashboard, nurses will be prompted to perform pressure ulcer preventative care, namely patient turning with satisfactory tissue decompression for 15 min, at least every 2 h (optimal). If a patient does not receive the full time of tissue decompression, the Leaf Patient Monitoring System automatically adjusts, proportionally reducing the time-to-next-turn. For example, if the patient was on their back and moved to their right side, but returned to their back within 7–8 min, the time-to-next-turn will be adjusted reciprocally from 2 h to 1 h. This ensures that patients receive at least 15 min of tissue decompression every 2 h.

If the subject is enrolled in the control group, the unit secretary will turn the User-Dashboard “off” by selecting the “control patient” check-box in the Leaf Patient Monitoring System. This will turn the sensor information off and therefore not display any information to the User-Dashboard. Patients in the control group will continue to receive standard care practices, that is, pressure ulcer-prevention activities initiated by nurses using their usual care routines. Nursing care will not be “optimized” by the Leaf Patient Monitoring System, with the system withholding any data related to turning frequency, position schedule, and decompression time. The patient sensor will continue to collect these data for the purposes of research analysis only.

Each patient’s participation within the study begins within the first hour of arrival to a study unit and ends upon discharge or transfer from the study unit. Upon leaving the unit, the patient sensor will be removed and the patient will be discharged automatically from the Leaf Patient Monitoring System.

### Technology

**Fig. 4:**
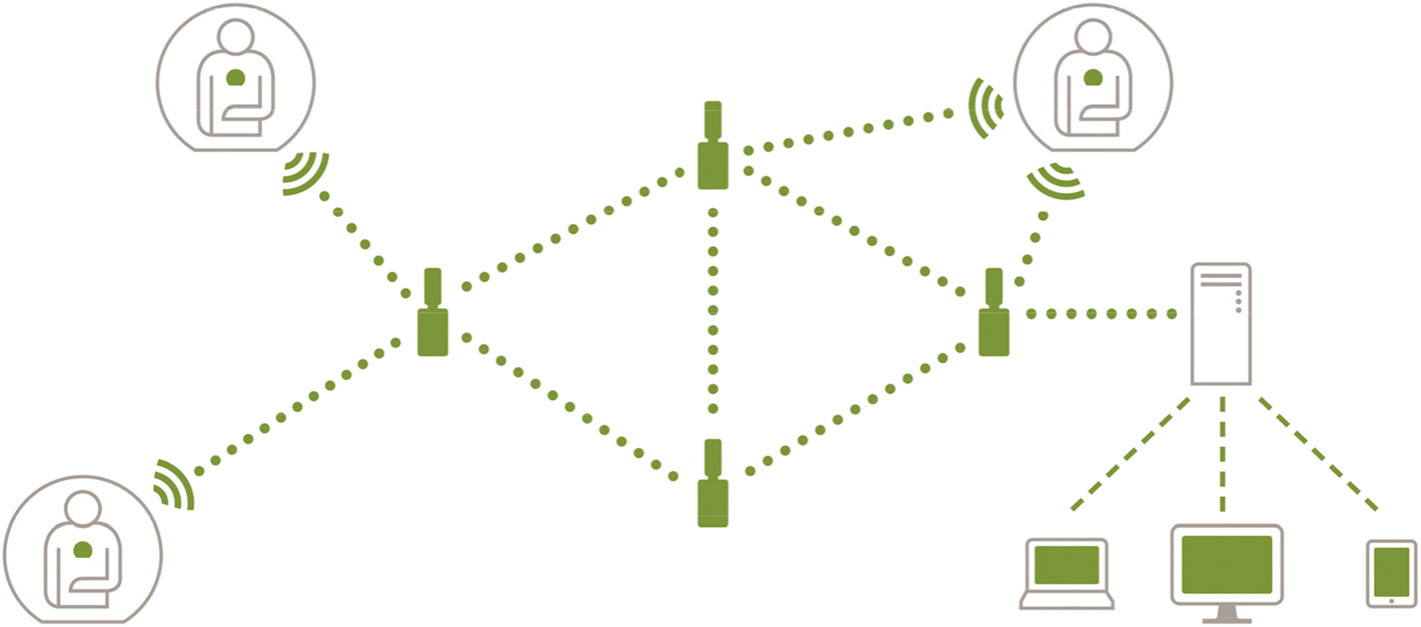
Wearable patient sensor communication network. *Used with permission from Leaf Healthcare, Inc.

The Leaf Patient Monitoring System is a proprietary system developed by Leaf Healthcare Inc. (Pleasanton, CA, USA) [10]. The Leaf Patient Monitoring System is a wireless monitoring system that enables personalized turning protocols. A wireless, wearable, single-patient sensor communicates a patient’s body position and movement, through a proprietary mesh network of relay antennas placed throughout the study units to a central monitoring system (Fig. 4). The Leaf Turn Management System accesses an industry standard Health Level Seven International (HL7)-ADT data stream. This allows for simplified and accurate patient enrollment and seamless tracking of patient movement throughout the study units. For data security, all data generated from the Leaf Patient Monitoring System are transferred and stored within the institution’s firewall to secure servers. The Leaf Turn Management System is the User-Dashboard that displays an individual patient’s positioning and movement data to the clinical team.

### Data management

Proprietary turning data will be acquired from the Leaf Patient Monitoring System. Compliance to preventative turning care will be defined by two measures. The first will sum the overall time the patient is overdue for turning care, divided by the total monitoring time, and will be reported as a percentile of time. For example, if a patient was on the unit for 4 h and did not receive preventative turning care at the second hour, but instead at the third hour (1 h overdue), then the patient’s care was in compliance for 3 of the 4 h of their length of stay. This would, therefore, represent a time in compliance of 75 %. If they received the turn on schedule, the compliance would be at 100 %. The second measure will divide a patient’s length of stay into 2 h turning blocks. In the above example, if the patient was turned once in their 4 h stay, compliance to turning would equal 50 %. Although we believe that the time in compliance is more appropriate than the proportion of turns within 2 h blocks, we will test and report on both measures as the latter reflects current clinical care practices.

### Pressure ulcer staging

The National Pressure Ulcer Advisory Panel (NPUAP) staging criteria will be used to stage all HAPUs [11]. Staging will be completed by an expert RN blinded to the patients’ study allocation. Adjudication will occur with the study team for any wounds that are difficult to stage.

- *Stage I, non-blanchable erythema*: intact skin with non-blanchable redness of a localized area usually over a bony prominence. Darkly pigmented skin may not have visible blanching; its color may differ from the surrounding area. The area may be painful, firm, soft, warmer or cooler as compared to adjacent tissue. Category I may be difficult to detect in individuals with dark skin tones. This stage may indicate “at risk” persons
- *Stage II, partial thickness skin loss*: partial-thickness loss of dermis presenting as a shallow, open ulcer with a red-pink wound bed, without slough. This may also present as an intact or open/ruptured serum-filled or sero-sanginous-filled blister. It presents as a shiny or dry, shallow ulcer without slough or bruising*. This category should not be used to describe skin tears, tape burns, incontinence-associated dermatitis, maceration or excoriation. *Bruising indicates deep tissue injury
- *Stage III, full-thickness skin loss*: full-thickness tissue loss. Subcutaneous fat may be visible but bone, tendon or muscle are *not* exposed. Slough may be present but does not obscure the depth of tissue loss. This *may* include undermining and tunneling. The depth of a category/stage III pressure ulcer varies by anatomical location. The bridge of the nose, ear, occiput and malleoli have no (adipose) subcutaneous tissue and category/stage III ulcers can be shallow. In contrast, areas of significant adiposity can develop extremely deep category/stage III pressure ulcers. Bone/tendon is not visible or directly palpable
- *Stage IV, full-thickness tissue loss*: full-thickness tissue loss with exposed bone, tendon or muscle. Slough or eschar may be present. This stage often includes undermining and tunneling. The depth of a category/stage IV pressure ulcer varies by anatomical location. The bridge of the nose, ear, occiput and malleoli have no (adipose) subcutaneous tissue and these ulcers can be shallow. Category/stage IV ulcers can extend into muscle and/or supporting structures (e.g., fascia, tendon or joint capsule) making osteomyelitis or osteitis likely to occur. Exposed bone/muscle is visible or directly palpable

### Data collection

Patient demographics and clinical data will be obtained from the Stanford Translational Research Integrated Database Environment (STRIDE). STRIDE is a clinical data warehouse that is maintained by the Stanford University School of Medicine and is updated in near real time directly from the EMR used at Stanford Health Care. All data generated from the study will be managed and stored using REDCap electronic data capture tools, hosted by Stanford University. REDCap (Research Electronic Data Capture) is a secure, web-based application designed to support data capture for research studies [12].

### Study endpoints

#### Primary

The following endpoints will be evaluated:

- Difference in compliance rates with preventative turning practices between the treatment and control groups, as continuously measured by the Leaf Patient Monitoring System during the patients’ ICU stay
- Difference in the proportion of HAPUs between the treatment and control groups

#### Secondary

Secondary measures are to:

- Evaluate and describe, any differences identified in hospital-acquired pressure ulcer rates and stages between medical and surgical patient groups
- Develop explanatory models of contributing clinical factors to HAPUs for the three groups: all ICU patients, medical patients only, and surgical patients only

### Sample size calculation

Using a two-sided *Z* test of the difference between proportions with 80 % power and a 5 % significance level, a sample size of 1812 patients, 906 in each group, will be sufficient to detect a clinically important difference of 50 % between the groups in the rate of HAPUs. This assumes that a 50 % difference represents a change from a prevalence rate of 5 % for pressure ulcers in the control group, to a rate of 2.5 % for pressure ulcers in the treatment group. Conservatively, with an estimated enrollment of 200 subjects per month, study enrollment is expected to be between 7 and 10 months’ duration.

### Statistical analysis

Data analysis will be conducted using IBM SPSS Predictive Analytics Software (v20, Armonk, NY, USA), after data cleaning and organization within Excel 2010 (Microsoft, Seattle, WA, USA). A consultant statistician, external to the study team and trained in quantitative methods will oversee data analyses.

#### Descriptive analysis

Demographic and clinical characteristics will be summarized for each treatment group and reported. Frequencies will be determined for count data, such as the proportion of patients with pressure ulcers, and descriptive summaries will be used for continuous data, such as percentage of compliance time. Measures of central tendency (mean, standard deviation, median, minimum, and maximum values) will also be reported.

#### Primary endpoint

An independent-samples Student’s *t* test will be used to evaluate for differences in preventative turning compliance between the two groups. To test for differences in pressure ulcer rates between the treatment groups, a 2 × 2 table with chi-square statistic will be used. To further evaluate for differences in pressure ulcer rates between treatment groups over time, a Kaplan-Meier survival analysis with log-rank test will be used. For all analyses, tests are deemed to be significant if significance is less than *p* <0.05. Device-related pressure ulcers from catheters or tubes are generally unrelated to turning and are not included in this analysis.

#### Secondary endpoint

To test for differences in HAPUs between patients based on admitting service (medical or surgical), a Kruskal-Wallis rank test will be used. Stages of pressure ulcers (based on NPUAP staging criteria) will represent the ranks to be measured. A difference will exist if the significance level of the test statistic is *p* <0.05.

In creating explanatory models, multivariate logistic regression will be used. First, univariate testing of each variable in relationship to the endpoint (hospital-acquired pressure ulcer) will be performed. Only variables having a maximum significance with the endpoint of *p <*0.20 will be included in the multivariate model. Age and sex will be reintroduced to the models if they fail to meet the inclusion criteria and all other variables will be entered into the model in a forward stepwise fashion. This will be rerun until only significant variables remain. Three logistic regression models will be constructed: all ICU patients, only medical patients, and only surgical patients.

### Ethics approval

The study protocol was reviewed and approved by the Stanford University Institutional Review Board (IRB). Due to the study procedures and patient acuity, a waiver of authorization was granted for enrollment. This study is registered with ClinicalTrials.gov, NCT02533726; however, it is not open to public enrollment.

## Discussion

This study investigates the effect of optimal turning, defined as patient turning every 2 h with at least 15 min of tissue decompression, on reducing HAPUs. It compares outcomes in the treatment group with a control group. The treatment group consists of patients receiving clinical care that is optimized by the Leaf Patient Monitoring System (Leaf Health Care, Pleasanton, CA, USA). This system monitors patient positioning and advises when a patient turn is due, based on preset criteria, and allows for personalized care practices.

The strength of this study is its robust design. A randomized control trial is planned, which to our knowledge is the first of its kind to use a wearable patient sensor to quantify and establish optimal preventative care practices, in an attempt to determine whether these are effective in reducing HAPUs. Prior studies have used an assortment of technical solutions, including environmental and mattress sensors measuring body temperature, estimated body position, and surface compression [13]. However, these studies have consistently failed to assess outcomes: specifically, whether these approaches actually reduce HAPUs. In this study, any positive results will be of immediate benefit as much of the study’s methodologies maintain standard care practices. Therefore, positive findings will be readily translated into clinical practices.

However, there are important challenges in this study. The first challenge is maintaining group assignment. We plan to randomize patients across two ICUs, involving 58 beds and over 300 RNs. Over the course of the ICU length of stay a patient may receive care from many RNs. During the study period it is very likely that an individual nurse will care for patients in both treatment and control groups. Therefore, there is a risk that nurses will transfer and apply knowledge of optimal turning practices between groups. In addition, although nurses remain within the patient’s room for their entire shift, it is plausible that communication regarding prevention care practices occurs during breaks, or with nurses in adjacent patient rooms. To identify and account for potential bias, a small pilot study will be conducted prior to the primary study to establish baseline adherence rates. Twenty-five patients from each unit will receive unmarked sensors from the research team. Nurses will be masked as to the sensors’ true intent and the sensors will collect the same patient-positioning information as the primary study. These data will be used to compare data derived from the control group, to assess for any potential cross-over or observer bias effects.

Another challenge within this study is the number of software steps required to ensure accurate function, feedback and group assignment. The clinical care team receive data from the User-Dashboard about a patient’s position and time-to-next-turn. However, the system requires that the unit secretary accurately enters the correct group assignment. Also, due to the complexity of clinical software applications, accessing the User-Dashboard is a two-step process for staff. Staff must separately open the software application in addition to the EMR. Once activated, the software reduces the technical burden on clinical staff as the User-Dashboard accesses a HL7-ADT feed. This automatically provides complete patient demographic data and tracks patient location throughout their ICU length of stay, minimizing the need for staff to continually update this information.

To further ensure that clinical staff complete study procedures, daily monitoring of study units will occur. To ensure that unit secretaries are correctly assigning patients based on the computer-generated randomization schedule, a patient sticker will be applied to the back of the randomization card and placed in a secured box at the point of enrollment. On a regular schedule these data will be collected, stored, and cross-referenced with the patient allocation entered into the Leaf Patient Monitoring System.

Finally, this study brings together research personnel from both clinical and technical teams of a major academic health system, working together to implement a large clinical trial within a complex care environment. As organizations focus on improving clinical care environments, building efficiencies in clinical research and in adopting and testing innovative technologies is imperative in improving health care delivery and clinical outcomes for patients.

### Trial status

At time of submission this trial is open to enrollment.

## Abbreviations

ADT: Admission-Discharge-Transfer
HAPU: Hospital-Acquired Pressure Ulcer
HL7: Health Level Seven International
ICU: Intensive Care Unit
PU: Pressure Ulcer
EMR: Electronic Medical Record
RN: Registered Nurse
